# A Systems Pharmacology Approach to Determine Active Compounds and Action Mechanisms of Xipayi KuiJie’an enema for Treatment of Ulcerative colitis

**DOI:** 10.1038/s41598-017-01335-w

**Published:** 2017-04-26

**Authors:** Wei Yu, Zhihong Li, Fei Long, Wen Chen, Yurong Geng, Zhiyong Xie, Meicun Yao, Bo Han, Teigang Liu

**Affiliations:** 1 School of Pharmacy, Xinjiang Shihezi University, Xinjiang, 832002 China; 2 The first affiliated hospital, School of medicine, Shihezi university, Xinjiang, 832002 China; 3 College of pharmacy, Sun yat-sen university, Guangzhou, 510006 China; 4 Key Laboratory of Chinese Internal Medicine of Education, DongZhiMen Hospital, Beijing, 100070 China; 5 College of Traditional Chinese Medicine, Beijing University of Chinese Medicine, Beijing, 100029 China

## Abstract

Xipayi Kui Jie’an (KJA), a type of traditional Uygur medicine (TUM), has shown promising therapeutic effects in Ulcerative colitis (UC). Owing to the complexity of TUM, the pharmacological mechanism of KJA remains vague. Therefore, the identification of complex molecular mechanisms is a major challenge and a new method is urgently needed to address this problem. In this study, we established a feasible pharmacological model based on systems pharmacology to identify potential compounds and targets. We also applied compound-target and target-diseases network analysis to evaluate the action mechanisms. According to the predicted results, 12 active compounds were selected and these compounds were also identified by HPLC-ESI-MS/MS analysis. The main components were tannins, this result is consistent with the prediction. The active compounds interacted with 22 targets. Two targets including PTGS2 and PPARG were demonstrated to be the main targets associated with UC. Systematic analysis of the constructed networks revealed that these targets were mainly involved in NF-κB signaling pathway. Furthermore, KJA could also regulate the CD4 + CD25 + Foxp3 + Treg cells. In conclusion, this systems pharmacology-based approach not only explained that KJA could alleviate the UC by regulating its candidate targets, but also gave new insights into the potential novel therapeutic strategies for UC.

## Introduction

Ulcerative colitis (UC) is a non-specific chronic inflammatory disorder and associated with recurrent attacks that may last several months to years^1^. Characteristics of UC are symptoms of acute pain, vomiting, weight loss, diarrhea, and bloody stool^2^, it is classified by the World Health Organization (WHO) as a refractory disease^3^. Agents that are commonly used to treat UC include 5-ASA, SASP, steroid hormones, anti-TNF-α drugs and immunosuppressive agents. Most of these therapies have side effects or are costly^4^. Therefore, the discovery of cost effective and efficacious agents and therapeutic methods for treating UC is necessary.

Traditional Uygur medicine (TUM) is an ethnic medical system. It is also broadly categorized as an alternative medicine. Some traditional TUM drugs have shown promising therapeutic effects for the treatment of UC^5^. Xipayi Kui Jie’an (KJA) is a prescribed medicine within the TUM approach. It is known as the “Xipayi gingiva protective solution” in the 1998 edition of the Ministry of Health of the People’s Republic of China Pharmaceutical Standards – Uyghur Medicine^6^. It is derived from Turkish galls, an insect gall that is produced when the larva of *Cynips gallae–tinctoriae* Oliv. parasitizes the tree branch of *Quercus infectoria* Oliv^7^. Turkish galls contain 50–70% gallotannin, and small amounts (2–4%) of gallic acid and ellagicacid. According to recent reports, in a rat model of UC, KJA was curative and showed a process of colon tissue morphology and pathological change. In this model, it was determined that treatment with KJA reduced inflammation, when the curative effect was evaluated after treatment^8–10^. In recent years, the use of KJA for the treatment of patients with UC has shown efficacy in the clinic. However, the complication in chemical composition and therapeutic targets of herbal medicines presents challenges in pharmacological investigations of KJA, which calls for a method that could decipher the relationships between the KJA and UC.

**Figure 1:**
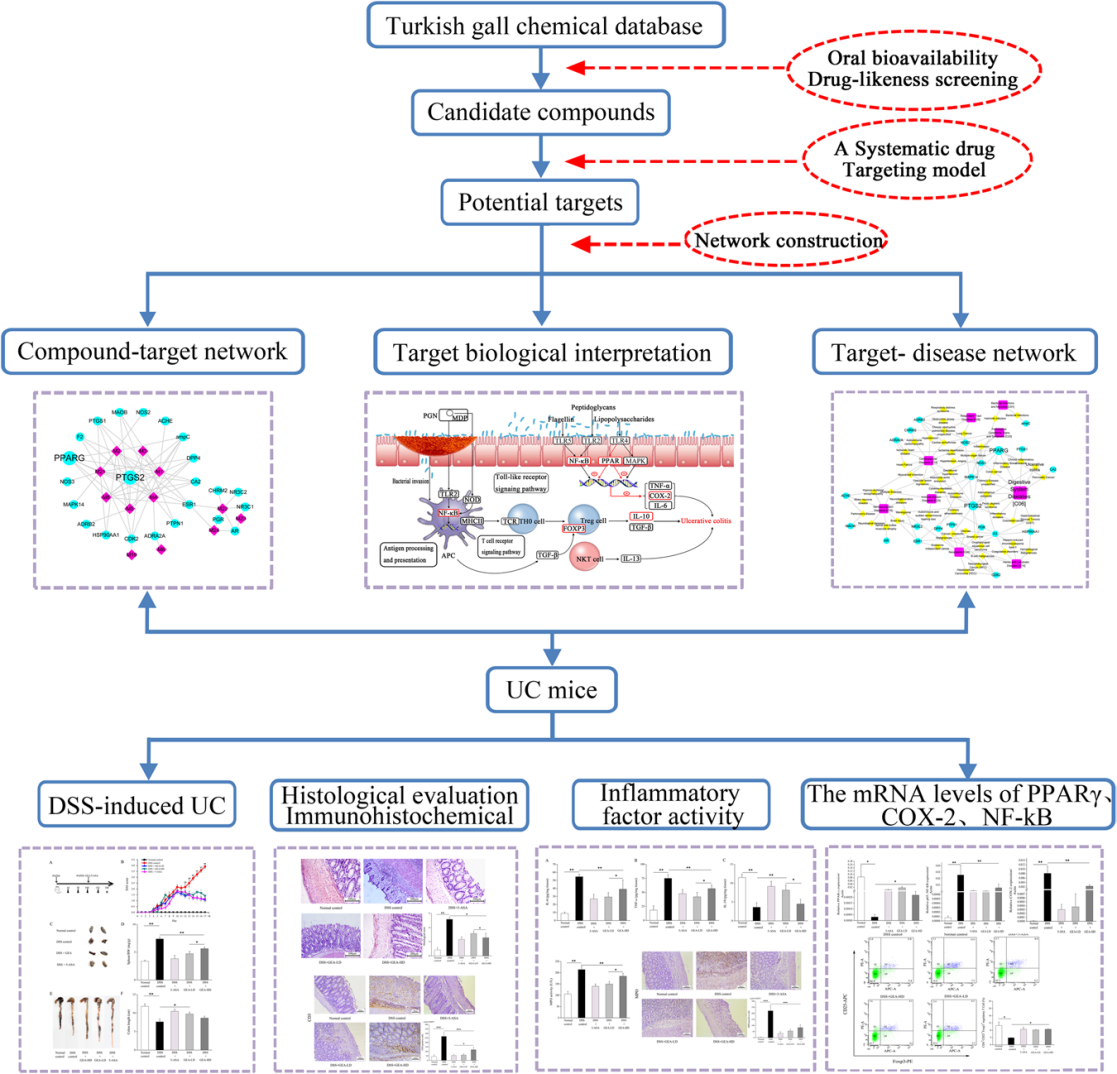
Systems pharmacology approach framework.

Systems biology analysis method is now perceived as an integral and efficient tool to study the role of TUM. Combined with pharmacology and pharmacodynamics, system biology has given birth to a promising subject, i.e., system pharmacology, will help to enhance the understanding of the complex molecular mechanisms underlying UC treatments. A systems pharmacology approach was used to investigate the pharmacological mechanisms of KJA in this study. A flowchart of the systems pharmacology approach is shown in Fig. 1. Active compounds in KJA was screened by *in silico* ADME system and predicted the potential related targets of these compounds by weighted ensemble similarity method. The obtained targets were mapped onto relevant databases to find out their corresponding pathways. following experiments were conducted in order to confirm whether the presumptive results of systemic pharmacology are correct^11,12^.

## Methods

### Turkish galls compound library construction

A total of 27 compounds, including 15 tannins, 2 flavonoids, 3 triterpenes, 2 polyphenols, and 5 phenolic acids, were collected as Turkish galls using literature published in the previous 32 years that was submitted to the Traditional Chinese Medicine Systems Pharmacology (TCMSP) database. Gallic tannin refers to gallic acid combined with polyol ester, usually of a molecular weight of 500–3000. Gallic tannin exists widely in the plant kingdom. Tannins is made up of gallic acid and many types of polyols such as glucose, hamamelis, quinic acid, fructose, and polygalitol. Gallic tannin can be easily hydrolyzed to gallic acid and polyol in the presence of base, acid, or enzyme. Many compounds can be downloaded from the TCMSP database.

### Dataset construction

As the chemical composition for Turkish galls is not included in the TCMSP database, we used references published over the last 10 years to obtain the chemical composition and then searched for corresponding targets and diseases from the database. Taking chemical composition as keyword, targets, disease-related data searched from TCMSP constitutes a database in our research.

### Active compound screening

#### Oral bioavailability prediction

Oral bioavailability (OB) is clearly an important pharmacokinetic parameter for orally administered drugs, indicating the degree to which a given compound may be delivered to the systemic circulation. In this study, in order to remove the compounds that are unlikely to become drugs, the OB values were calculated with a robust in-house tool (OBioavail1.1)^13^. Candidate compounds were those with OB ≥ 30%. The criteria used were as follows: (1) the extraction of as much information as was possible from the herbs could be affected with the least number of components, and (2) a reasonable explanation of the obtaining model was possible from the reported pharmacological data^14^.

#### Drug-likeness prediction

Drug-likeness (DL) is a concept that is used to assess how “drug-like” a prospective compound is. This assists in optimizing pharmacokinetic and pharmaceutical properties, such as solubility and chemical stability^15^. The definition of the TS index is: The Tanimoto coefficient^16^ is employed to filter out compounds that are seen as chemically unsuitable for drugs. The TS index was introduced to describe how herbal compounds are similar to known drugs in the Drug-bank database (http://www.drugbank.ca/). The TS index is defined as follows:

1 $${TS}({x},{y})=\frac{xy}{{\Vert x\Vert }^{2}{\Vert y\Vert }^{2}-xy},$$

where *x* is the molecular descriptor of the herbal compounds, and *y* is the average molecular properties of all molecules in the Drug-bank database. In this study, the DL ≥ 0.18 was used to select candidate compounds for further research.

### Target prediction

Using integration of chemical, genomic, and pharmacological data, was achieved with two powerful methods; i.e., Random Forest (RF) and Support Vector Machine (SVM)^17^, to predict the potential target profiles of active compounds. These two models allowed the prediction of drug-target mutual effects with a concordance rate of 82.83%, sensitivity of 81.33%, and specificity of 93.62%. Herbal ingredients with RF ≥ 0.7 or SVM ≥ 0.8 were chosen as potential active compounds in this study.

### Network construction

Networks can be used to view global relationships between nodes. Compound-target (C-T) and target-disease (T-D) networks were used to identify potential drugs and their corresponding mechanisms based on the compound-target-disease association (CTDA) method using Cytoscape 2.8.1^18^. A C-T network was generated by linking the screened candidate compounds and their potential targets. The T-D network was constructed by relating relevant targets to their diseases. The potential diseases were obtained from the Therapeutic Targets Database (TTD; http://bidd.nus.edu.sg). In graphical networks, nodes represent compounds, proteins or diseases and edges represent the compound–target or target–disease interactions.

### Pathway construction and analysis

At the pathway level, to probe into the action mechanisms of the formula for UC, an incorporated “UC pathway” was established based on current knowledge of UC pathology. First, we obtained the details of the human target proteins and then they were inputted into the Kyoto Encyclopedia of Genes and Genomes (KEGG, http://www.kegg.jp/) database, to acquire pathway information. Then, based on this basic pathway information, we assembled an incorporated UC pathway by picking out closely linked pathways related to UC pathology.

## Experimental

### Materials and Methods

Acetonitrile (HPLC grade) was purchased from Fisher Scientific (Fair Lawn, NJ, USA) and the formic acid (Analytical grade) from Tianjin Guangfu Fine Chemical Research Institute (Tianjin, China). Ultra-pure water was prepared by Milli-Q Integral water system (Millipore, Bedford, USA).

Turkish galls was identified by associate professor Bo Han in school of pharmacy, Xinjiang Shihezi University.

### Sample preparation

Turkish galls powder 100 g was accurately weighed and mixed with 1000 mL distilled water and subjected to reflux extraction for 3 times, the water extraction liquid was filtered and concentrated in vacuum. The product was extracted by diethyl ether and ethyl acetate and concentrated to a viscous liquid, this liquid was dissolved in distilled water and filtered, then transferred 1.0 mL of the solution carefully and completely to the volumetric flask, diluted with distilled water to volume, and mixed. The final concentration of samples of Turkish galls were 2 mg·mL^−1^. The sample was further filtered through a 0.22 μm filter membrane for HPLC-ESI-MS/MS determination.

### Instrumentation and chromatographic conditions

The HPLC-MS analyses were carried out using a quaternary solvent manager, sample manager with flow-through needle system and an online degasser. Chromatographic separation was performed on a Sun Fire C18 column (Φ 4.6 mm × 150 mm, 5 μm; Waters, USA). The sample was eluted at a flow rate of 1 mL·min^−1^ in a gradient elution program of A (0.2% formic acid: water) and B (acetonitrile): 0–8 min (90–80% A); 8–35 min (90–80% A); 35–50 min (80–70% A); 50–60 min (70–0% A). The injection volume was 20 μL. Mass spectrometry was carried out using a QDA system (Waters, The United States) equipped with an electrospray ionisation (ESI) source, negative ionization mode was used for MS analyzes.

Mass spectrometry was carried out using a XEVO TQD system (Waters, The United States, Massachusetts) equipped with an electrospray ionisation (ESI) source. The operating parameters were optimised as follows: capillary voltage, 2.64 kV; desolvation temperature, 350 °C; source temperature, 150 °C; desolvation gas (N2), 800 (L/Hr) and collision gas, argon. Negative ionization mode was used for MS/MS analyzes, which was helpful for the determination of elemental composition by accurate mass measurement.

### Animals and experimental design

#### Animals

Kunming mice (clean grade, male, 6–8 weeks old, 30–35 g) were supplied by the Experimental Animal Research Center in Xinjiang (SCXK (xin) 2011–0001). The Animal Care Committee of the First Affiliated Hospital of the Medical College, Shihezi University approved all the experiments. Research was performed in accordance with relevant guidelines and regulations. All efforts were made to minimize animal suffering. All mice were housed in a controlled environment (temperature, 22 °C ± 2 °C; humidity, 50% ± 10%; lights, 12 h light/dark cycle). Animals were acclimatized to the environment for 1 week with free access to a standard pellet diet, before initiation of the experiments. Animals were randomly divided into 5 groups of 6 animals per group: a normal control group, a DSS control group, the 5-aminosalicylic acid group (5-ASA; Sigma-Aldrich Chemical Co., USA) at a dose of 50 mg/kg (5-ASA), a low-dose Turkish galls ethyl acetate extraction part-treated group (0.235 mg/g) (GEA-LD) group, and a high-dose Turkish galls ethyl acetate extraction part-treated (0.476 mg/g) (GEA-HD) group.

#### Induction of colitis

DSS (36 000–50 000 MW; MP Biomedicals, USA) was dissolved in distilled water at a concentration of 4%, and given to mice as drinking water from the first experimental day, to induce colitis. The normal control group comprised mice drinking distilled water without DSS.

After consumption of 4% DSS for 7 days, more than 90% of the DSS-receiving mice excreted formless and bloody feces. Mice showing bloody, formless feces, but not >3 g decrease in bodyweight on any serial days were selected as colitis mice. Mice were allocated to 4 groups consisting of 6 animals each: a DSS control group, a 5-ASA group, a GEA-LD group, and a GEA-HD group. The mice were dosed using enema suspensions administered rectally (by inserting the enema tube into the rectum approximately 4 cm) in the morning or late afternoon for 10 days. The normal group and DSS control group were administered saline by a similar route.

A disease activity index (DAI) was calculated. The following parameters were graded on a scale of 0 to 4: loss of body weight (BW) (0, no loss; 1, 0–5%; 2, 5–10%; 3, 10–20%; 4, >20%), stool consistency (0, normal; 2, loose stools; 4, watery diarrhea) and the occurrence of gross blood in the stool (0, negative; 4, positive)^19^.

### Histological evaluation of colitis

At the end of the experimental period the animals were killed by cervical dislocation. The spleen was dissected out and weighed. The whole colon from the cecum to the anus was removed and then fixed in 10% buffered formalin that was embedded in paraffin and stained with H&E to Histological evaluation of colon. The histological inflammatory scored were from 0 (normal) to 10 (severe colitis), which were determined according to previously described method^20^. A total scoring system was based on 3 parameters: mucosal ulceration, 0–3 (0, normal; 1, surface epithelial inflammation; 2, erosions; 3, ulcerations); epithelial hyperplasia, 0–3 (0, normal; 1, mild; 2, moderate; 3, pseudopolyps); and lamina propria mononuclear infiltrate, 0–2 (0, normal; 1, slightly increased; 2, markedly increased).

### Immunohistochemistry (IHC)

To evaluate the severity of DSS-induced UC on day 17, The intensity of inflammation was determined using anti-MPO polyclonal antibody (ab9535, Abcam, United Kingdom). anti-CD3 antibody (ab16669, Abcam, United Kingdom) The number of myeloperoxidase-positive neutrophils, CD3-positive T lymphocytes in 10 randomly selected fields of inflamed mucosal areas per section (original magnification: ×200).

### Enzyme-linked immunosorbent assay (ELISA) for TNF-α, IL-6, IL-10

The colon tissues were rinsed in ice-cold PBS to remove excess blood. Then they were weighed before homogenization. The tissues were cut up into small pieces and then homogenized in PBS (w:v = 1:20–1:50). Then the homogenates were centrifuged for 5 min at 10,000× g. The resulting supernatant was used to estimate TNF-α, IL-10, and IL-6 using mouse-specific ELISA kits (USCN Life Science & Technology Company, USA), per the manufacturer’s instructions.

### Isolation of spleen lymphocytes and flow cytometry

At the end of 17 days of the experiment, the suspensions of single cells were prepared from the mice spleens, which were mechanically dissociated and then filtered through nylon membranes. The single cell suspensions was carefully spread on lymphocyte separation medium and centrifuged at 400× g for 20 min. The lymphocyte separation medium was carefully extracted and added to the cell-washing liquid, and centrifuged at 250× g for 10 min. The precipitation was re-suspended with PBS for flow cytometry assay.

Lymphocytes were isolated from the SPs. They were then stained with FITC-conjugated anti-CD4, APC-conjugated anti-CD25 and PE-conjugated anti-Foxp3 with a staining kit (88.8111, eBioscience, USA) according to the manufacturer’s instructions. The quantity of CD4^+^CD25^+^Foxp3^+^Treg cells were calculated using flow cytometry assay.

### Quantitative Real-time PCR analysis

The mRNA expression of predicated Turkish galls targets, peroxisome proliferator-activated receptor (PPAR)-γ, cyclooxygenase (COX-2), and nuclear factor κB (NF-κB) pathway was validated by Quantitative Real-time PCR (qRT-PCR) analysis. TRIzol extraction reagent (15596026, ThermoFisher Scientific, USA) was used to isolate the total RNA of colon pieces according to the manufacturer’s instructions. Subsequently, Complementary DNA (cDNA) was reverse-transcribed from 1 μg RNA using a cDNA synthesis kit (K1622, ThermoFisher Scientific, USA). Quantitative RT-PCR was carried out using SYBR^®^ Select Master Mix (1602041, Life-Applied Biosystems, USA). The primer sequences were as follows: PPAR-γ sense: 5′-GTGATGGAAGACCACTCGCATT-3′, antisense: 5′-CCATGAG GGAGTTAGAAGGTTC-3′; COX-2 sense: 5′-GCCCAGCACTTCACGCATCAG-3′, antisense: 5′-GACCAGGCACCAGA CCAAAGACC-3′; p65-NF-κB sense: 5′-TGGC GAGAGAAGCACAGATA-3′, anti sense: 5′-TGTTGGTCTGGATTCGCTG-3′; β-actin sense: 5′-GGCGGACTGTTAC TGAGCTG-3′, antisense: 5′-CTGCGCAAGTT AGGTTTTGTCA-3′.

### Statistical analysis

Statistical software of the SPSS 22.0 was used to analyze the animal experiment data, the data were compared using the Student’s t-test or one-way analysis of variance for statistical significance of differences between or among different treatments. The data were expressed as the means ± SD, P < 0.05 was considered statistically significant.

## Results

### Active compound screening

**Figure 2:**
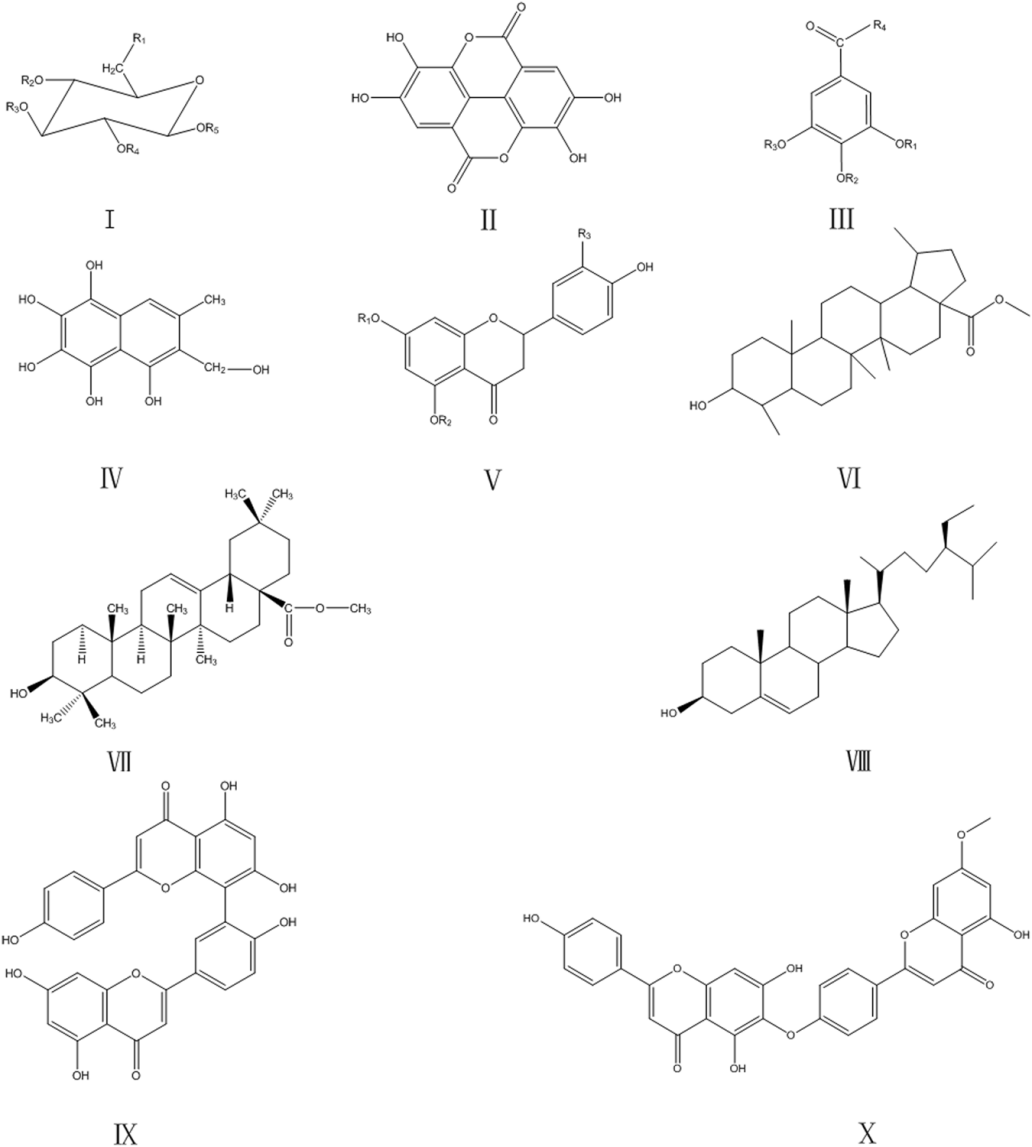
Turkish galls: 10 types of bone structure compound.

**Figure 3:**
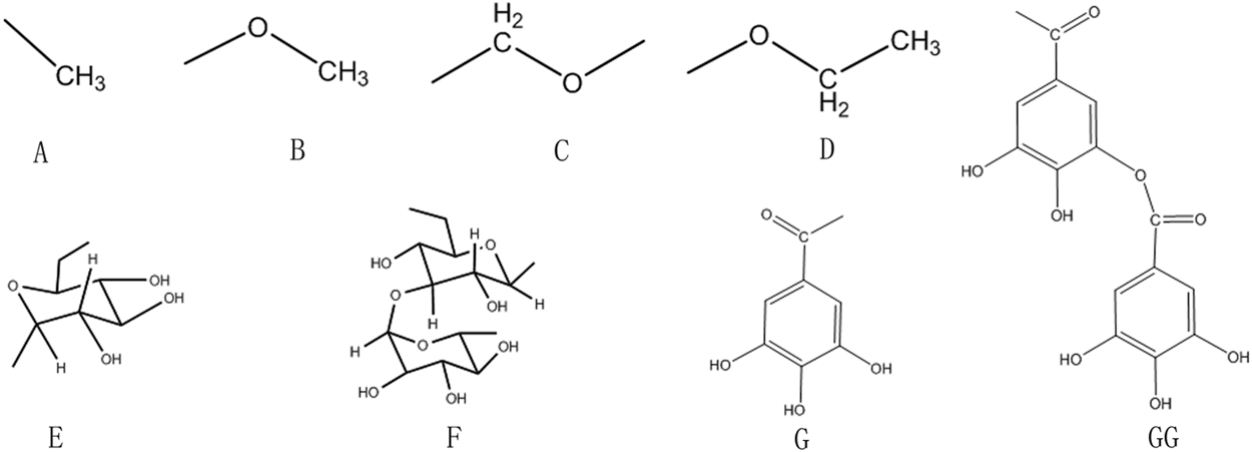
Turkish galls: chemical composition of common substituents.

We manually collected 27 compounds from Turkish galls, and used OB and TS evaluation to eliminate the ineffective compounds. OB is an important pharmacokinetic parameter of ADME processes. It involved several factors, such as gastrointestinal absorption and transit and chemical stability. High OB is an important consideration as a therapeutic agent in the development of molecules. Therefore, OB screening was carried out to determine orally available drug candidates. TS is an additional important drug research parameter that be used to evaluate whether a compound functions as a drug. Compounds that pass OB and TS screening are considered pharmacologically active. In our study, 12 compounds were found to have satisfactory OB (OB > 30%) and desirable TS properties (TS > 0.18), or significant activity. Most screened compounds had both high OB and DL values. Representative active compounds that exhibited anti-inflammatory activities were screened out, including p-digallic acid, m-digallic acid, gallic acid, and gallic acid ethyl ester. Interestingly, the selected compounds from Turkish galls were found to skeleton with a simple side chain (Fig. 2). The chemical composition of the common substituents of Turkish galls is shown in Fig. 3 and further details are provided (Tables 1–4). According to Tables 1–4, the most compounds of Turkish galls were tannis.

**Table 1:** Type I Turkish galls compounds.

ID	Compound	Nuclear	R_1_	R_2_	R_3_	R_4_	R_5_	References
1	1-O-galloyl-O-β-D-glucose	I	H	H	H	H	G	22
2	1,2,6-tri-O-galloyl-β-D-glucose	I	OG	H	H	G	G	22
3	1,6-di-O-galloyl-O-β-D-glucose	I	OG	H	H	H	G	22
4	1,2,3,6-tetra-O-galloyl-β-D-glucose	I	OG	H	G	G	G	22
5	1,2,3,4,6-five-O-gallic acid-β-D-glucose	I	OG	G	G	G	G	22
6	6-O-digalloyl-1,2,3-three-O-galloyl-β-D-glucose	I	OGG	G	G	G	G	22
7	2-O-digalloyl-1,3,4,6-four-O-gallic acid-β-D-glucose	I	OG	G	G	GG	G	22
8	3-O-digalloyl-1,2,4,6-four-O-gallic acid-β-D-glucose	I	OG	G	GG	G	G	22
9	4-O-digalloyl-1,2,3,6-four-O-gallic acid-β-D-glucose	I	OG	GG	G	G	G	22
10	2,6-Bis-O-digalloyl-1,3-two-O-galloyl-β-D-glucose	I	OGG	H	G	G	G	22
11	6-O-digalloyl-1,2,3,4-four-O-galloyl -β-D-glucose	I	OGGG	H	G	G	G	22
12	6-O-digalloyl-1,2,3,4-four-O-galloyl -β-D—glucose	I	OG	G	G	G	G	22

**Table 2:** Type II Turkish galls compounds.

ID	Compound	Nuclear	R_1_	R_2_	References
13	Ellagic acid	II	H	H	23
14	Ellagic acid-4-O-[-D-giucopyranosyl] -10-O-[-D-giucopyranosyl]-(4 → 1)-β-D-rhamnopyranoside	II	E	F	24

**Table 3:** Type III Turkish galls compounds.

ID	Compound	Nuclear	R_1_	R_2_	R_3_	R_4_	References
15	Gallic acid ethyl ester	III	OH	OH	OH	C	21
16	Clove acid	III	B	OH	B	OH	21
17	P-digallic acid	III	OH	G	OH	OH	21
18	Methyl gallate	III	OH	OH	OH	B	21
19	Gallic acid	III	OH	OH	OH	OH	21
20	M-digallic acid	III	OH	OH	G	OH	21

**Table 4:** Other compounds.

ID	Compound
21	2-methyl-3-hydroxymethylene-4,5,6,7-pentahy-droxynathalene
22	Methyl betulate
23	Methyl oleanolate
24	β-sitosterol

### Network construction and analysis

#### C-T network

**Figure 4:**
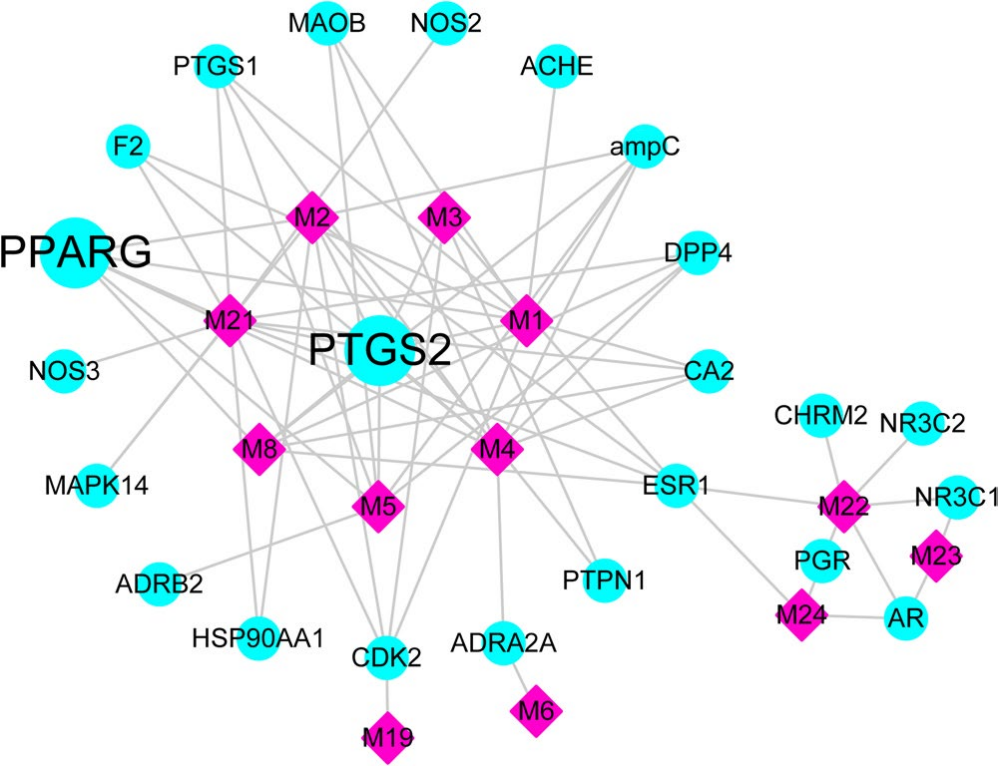
C-T network of Turkish galls. A protein node and compound node are linked if the protein is targeted by the corresponding compound. The node size is proportional to its degree. The dark pink diamonds represent the Turkish galls compounds. The light green circles represent the Turkish galls targets.

TUM exerts extensive biological and pharmacological effects through multiple compounds and targets. To understand the complex interaction of compounds and their corresponding targets at a systems level, we constructed a C-T network based on the candidate compounds of Turkish galls and the potential targets. The C-T network embodied 35 nodes (27 compounds, 12 candidate compounds, and 22 potential targets) and 68 C-T interactions (Fig. 4). The mean degree value (the number of associated targets) of candidate compounds was 5.8, and 7 compounds had a degree value >6, indicating that most of the compounds regulated multiple targets to exert various therapeutic effects. Specifically, 3 compounds, 2-methyl-3-hydroxymethylene-4,5,6,7-pentahy-droxynathalene, 1-O-galloyl-O-β-D-glucose, and gallic acid, acted on 14, 10, and 9 targets, respectively, meaning they are crucial active compounds of Turkish galls because of their important positions in this network. Additionally, the results indicated that many targets were hit by multiple compounds in the C-T network. For example, PTGS2 and PPAR-γ were targeted by 7 and 6 compounds, respectively. PPAR-γ, or NR1C3, is a member of the nuclear hormone receptor family. It plays a central role in adipocyte differentiation and insulin sensitivity and is a critical regulator of the inflammatory response^25–27^. Evidence has suggested that PPAR-γ plays a key anti-inflammatory role during intestinal inflammation. PPAR-γ ligands have inhibited inflammation and reduced disease severity in various experimental models of colitis^28–31^. In patients with mild to moderate UC, administration of PPAR-γ agonists such as thiazolidinediones and 5-aminosalicylic acids (5-ASA) has induced and maintained clinical remission^32–34^. Meanwhile, another candidate target, COX-2, has been shown to produce prostaglandins (PGs) from arachidonic acid, which is associated with mediation of inflammation. In addition, it has been reported that PGE2 and COX-2 expression levels are elevated in inflamed mucosal tissues of patients with UC^35,36^.

#### T-D network

**Figure 5:**
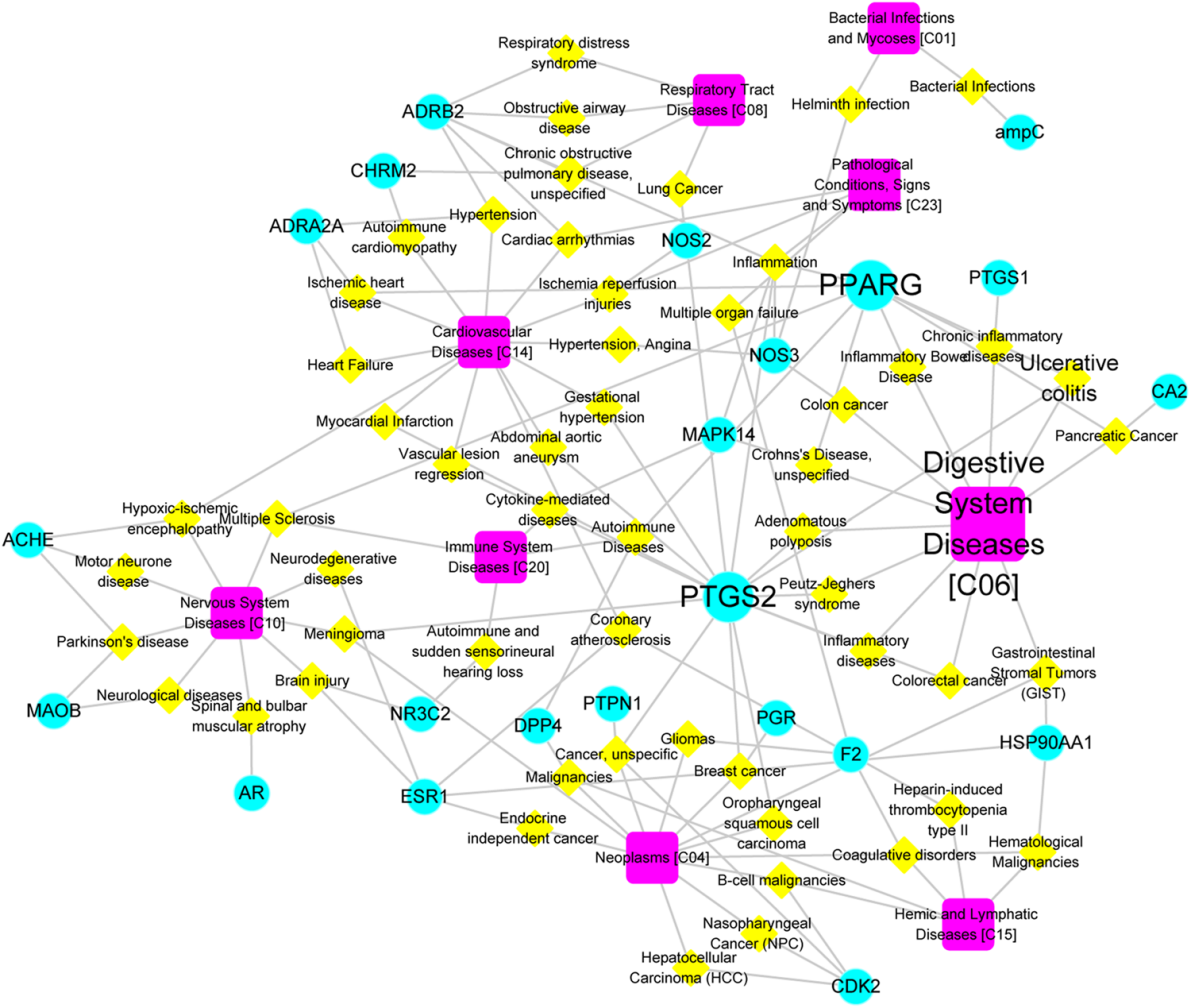
T-D network of Turkish galls. The T-D network is built by a target and diseases. Node size is proportional to its degree. The light green circles indicate the targets of Turkish galls, the yellow diamonds indicate the diseases that may be treated using Turkish galls, and the pink rounded rectangles indicate the disease types.

To gain better insight into the diseases that could be modified by Turkish galls, the potential targets were projected to Drug Bank and TTD databases to find their corresponding diseases. Fifty-five diseases were classified into 9 groups according to the MeSH Browser (2014MeSH). Finally, a T-D network was constructed based on potential targets and their corresponding diseases. Many of the identified diseases belong to neoplasms (9/55), or are nervous system (9/55) or digestive system diseases (13/55) (Fig. 5), implying that Turkish galls may be effective not only in the treatment of UC but also for these diseases. These results have supplied the systematic evidence for TUM theory, that a TUM formula has extensive pharmacological activity and may be successfully applied in the treatment of various diseases. For example, we found that PTGS2, an important target of Turkish galls, was associated with inflammation-associated diseases, such as inflammatory bowel disease, in the T-D network. Other studies have also suggested that PTGS2, as a type of anti-inflammatory drug target, is linked to the pathogenesis of these diseases, and is tractable as a new therapeutic target^37^. Based on these findings, Turkish galls contained numerous effective substances with different pharmacologic properties that may act on multiple targets with potential synergistic effects. To verify further the systems pharmacology predictions, experimental studies were conducted.

#### Pathway construction and analysis

**Figure 6:**
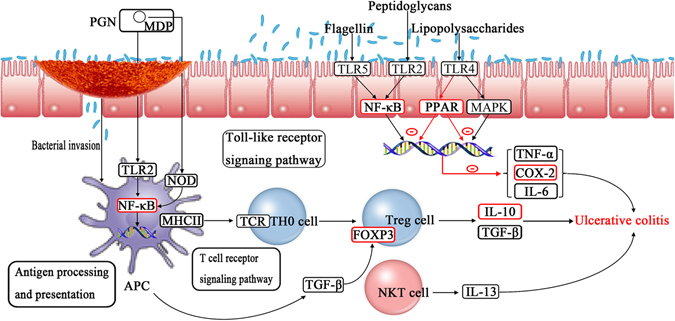
Distribution of Turkish galls target proteins on the compressed UC pathway. Induction of PPAR-γ expression in intestinal epithelial cells by lipopolysaccharide (LPS) activates TLR4, leading to regulation of NF-κB control of the inflammatory response. COX-2, which is downstream of the NF-κB pathway, shows a similar response to Turkish galls treatments. Also, there is a subsequent reduction in the expression of inflammatory factor IL-6 and TNF-α. Tregs play a crucial role in UC and they are capable of modulating effector T cells and antigen-presenting cells (APC) by secreting anti-inflammatory cytokines, such as IL-10.

To explore the integral regulation of KJA for the treatment of UC, we assembled an integrated UC pathway (Fig. 6) based on current knowledge of UC pathogenesis. The obtained human target proteins were input into the KEGG pathway database, and the results showed that 2 targets could be mapped according to the KEGG pathways. PPAR-γ, COX-2 targeted by Turkish galls were mapped onto a key UC process, these targets were mainly involved in NF-κB signaling pathway (Fig. 6), indicating that anti-inflammatory action may play a vital role in human diseases, especially inflammatory bowel disease^38–40^. The activated NF-κB pathway promotes the expression of various pro-inflammatory genes such as TNF-α, IL-6, and the pro-inflammatory enzyme COX-2, influencing the course of mucosal inflammation^41^. According to the pathway that we found, Turkish galls mitigate the severity of colitis by activating the expression of PPAR-γ, attenuating NF-κB activation and inhibiting the expression of pro-inflammatory cytokines and COX-2. In addition, we found that Turkish galls also regulated the immune cell, Tregs, and inhibited the expression of pro-inflammatory cytokines. These predictions suggest that Turkish galls has potential therapeutic value in the treatment of UC.

### Authenticate reverse results

#### Evaluation of the chemical properties of Turkish galls

**Figure 7:**
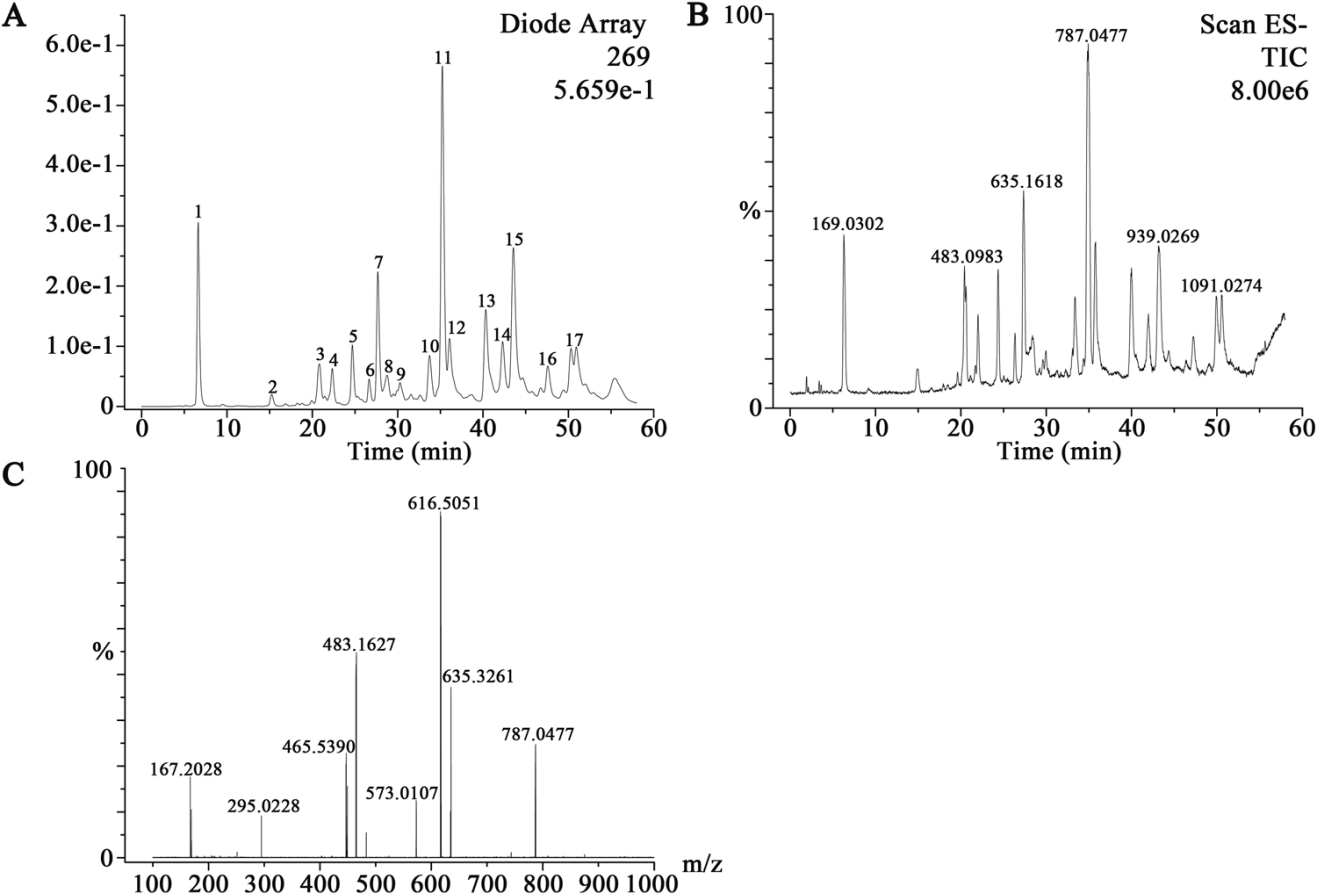
(**A**) HPLC analysis of Turkish galls ethyl acetate fraction. Peak assignment: (1) Gallic acid, (2)–(4) Digalloyl-glucoside, (5)–(9) Trigalloyl-glucoside, (10)–(12) Tetragalloyl-glucoside, (13)–(16) Pentagalloyl-glucoside peak, (17) Hexagalloyl-glucoside. (**B**) HPLC-MS analysis of Turkish galls ethyl acetate fraction. (**C**) HPLC-ESI-MS/MS data of m/z 787.0477, which identified as Tetragalloyl-glucoside.

HPLC-MS analysis of Turkish galls ethyl acetate fraction is shown in Fig. 2, according to our group of unpublished work, LC-MS was employed to identify the Turkish galls chemical composition. The identification processes are briefly described as follows, the loss of 169 Da MS/MS fragments from the precursor ion are identified as glucosyl units and the fragment of 152 Da and 170 Da fragments from the precursor ion are identified as galloyl moiety and galloyl units respectively. The Fig. 2A is HPLC (269 nm) spectrum and the Fig. 2B is HPLC-MS analysis of Turkish galls ethyl acetate fraction. By peak area normalization, the total peak area of 97.08% and these compounds are polyphenolic compounds, in which, the tannins was 88.14% and the phenolic acids was 8.94% (Fig. 7A). Take peaks 10, 11 and 12 as an example, which were identified as Tetragalloyl-glucoside with a [M-H]^−^ ion at m/z 787.0477 and fragments of m/z 635 and 617 in the MS^2^ experiment caused by the loss of a galloyl moiety (152 Da) and gallic acid (170 Da), respectively (Fig. 2C). The MS/MS spectrum of peaks gave the characteristic fragments at m/z 787.2259, m/z 635.3261, m/z 616.5051, m/z 483.1627, m/z 465.5390, m/z 295.0228, m/z 167.2028 respectively.

#### Turkish galls Ameliorates DSS-induced Acute Colitis in KM mice

**Figure 8:**
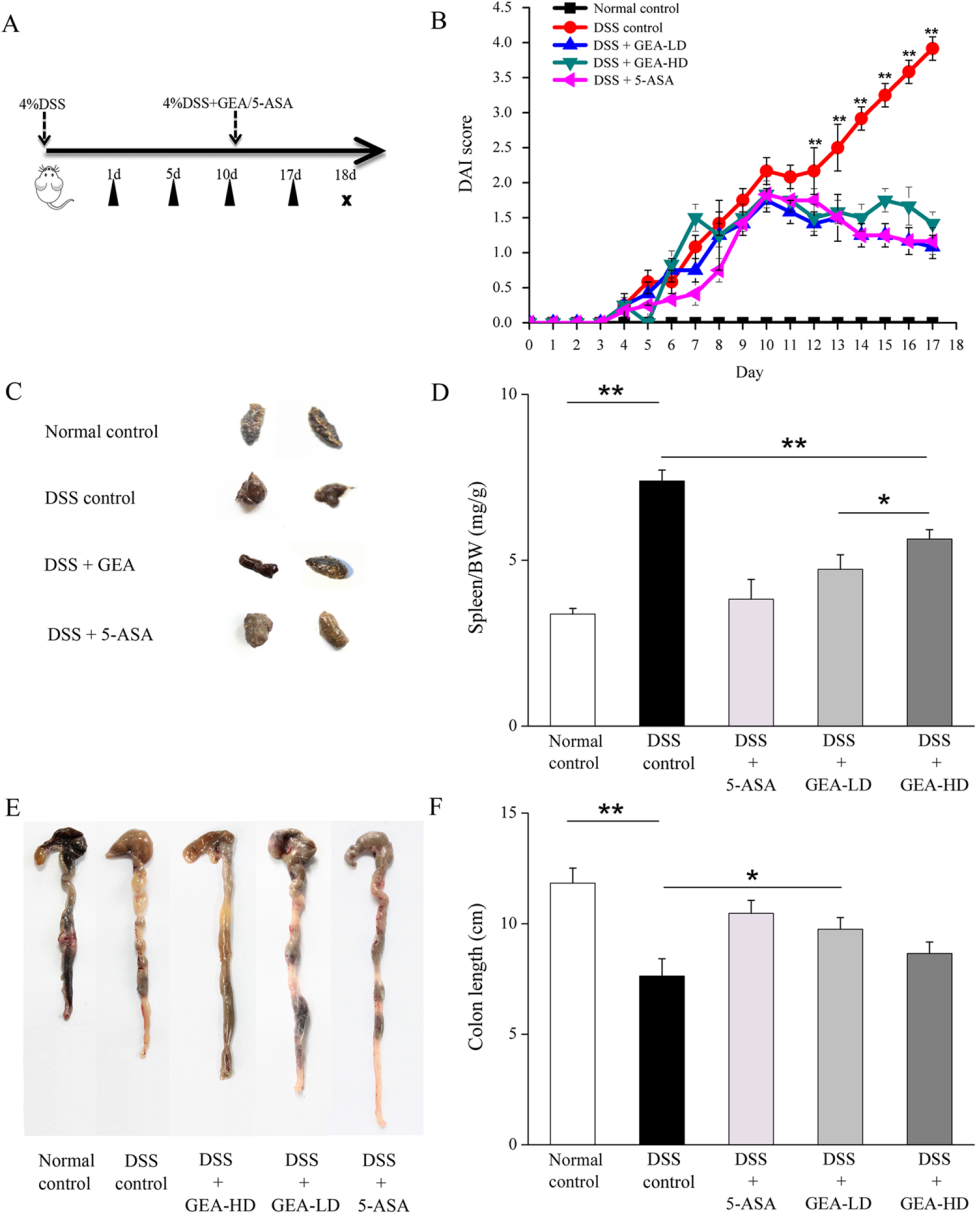
Turkish galls inhibits DSS-induced UC in mice. (**A**) Methods for DSS-induced colitis. (**B**) DAI was measured daily. (**C**) Representative photographs of feces. (**D**) Spleen/BW ratio was determined at the end of the experimental period. (**E**) Representative photographs of colon. (**F**) On day 17 of the experiments, the colon was removed, and the length was measured. The values are expressed as the mean ± S.D. *P < 0.05, **P < 0.01, ***P < 0.001.

Mice were followed by provision of 4% DSS for 17 days to induce colitis to confirm the curative effects of the Turkish galls extract. Mice in the DSS group quickly developed typical symptoms of clinical colitis, including diarrhea, gross blood and loss of body weight. These symptoms occurred at approximately day 10 after starting DSS administration. The methods with DSS-induced the colitis (Fig. 8A). The DAI scores are shown in Fig. 8B. The DSS control mice were dramatically increased from day 10 to 17 (day 17: DSS 3.92 ± 0.17 vs. normal 0 ± 0; P < 0.01) compared with the Normal control group, however, the DAI was found to be significantly lower in the 5-ASA group (1.25 ± 0.17), the GEA-LD group (1.08 ± 0.17) and GEA-HD group (1.42 ± 0.17) than that of mice treated with DSS alone (P < 0.01, respectively). Representative photographs of feces in the Fig. 8C. The ratio spleen/BW in the DSS control group was dramatically increased (DSS 7.40 ± 0.33 mg/g vs. normal 3.40 ± 0.20 mg/g; P < 0.01) compared with the Normal control group. In the 5-ASA (4.01 ± 0.20), the GEA-HD (5.72 ± 0.31 mg/g) and GEA-LD (4.70 ± 0.41 mg/g) groups the spleen/BW ratio was significantly suppressed on day 17 after DSS injury (P < 0.01, respectively; Fig. 8D). Correspondingly, shortening of the colon length is also considered to be a marker for colitis. The colon length was significantly shortened in the DSS group compared with control group (DSS 8.01 ± 1.44 cm vs. normal 12.00 ± 0.71 cm; P < 0.01). Strikingly, the GEA-LD group exerted preventive effects on the colon lengh of colitis mice (9.75 ± 0.29 cm; P < 0.05) (Fig. 8E,F).

#### Histology of DSS-induced Colitis in Mice

**Figure 9:**
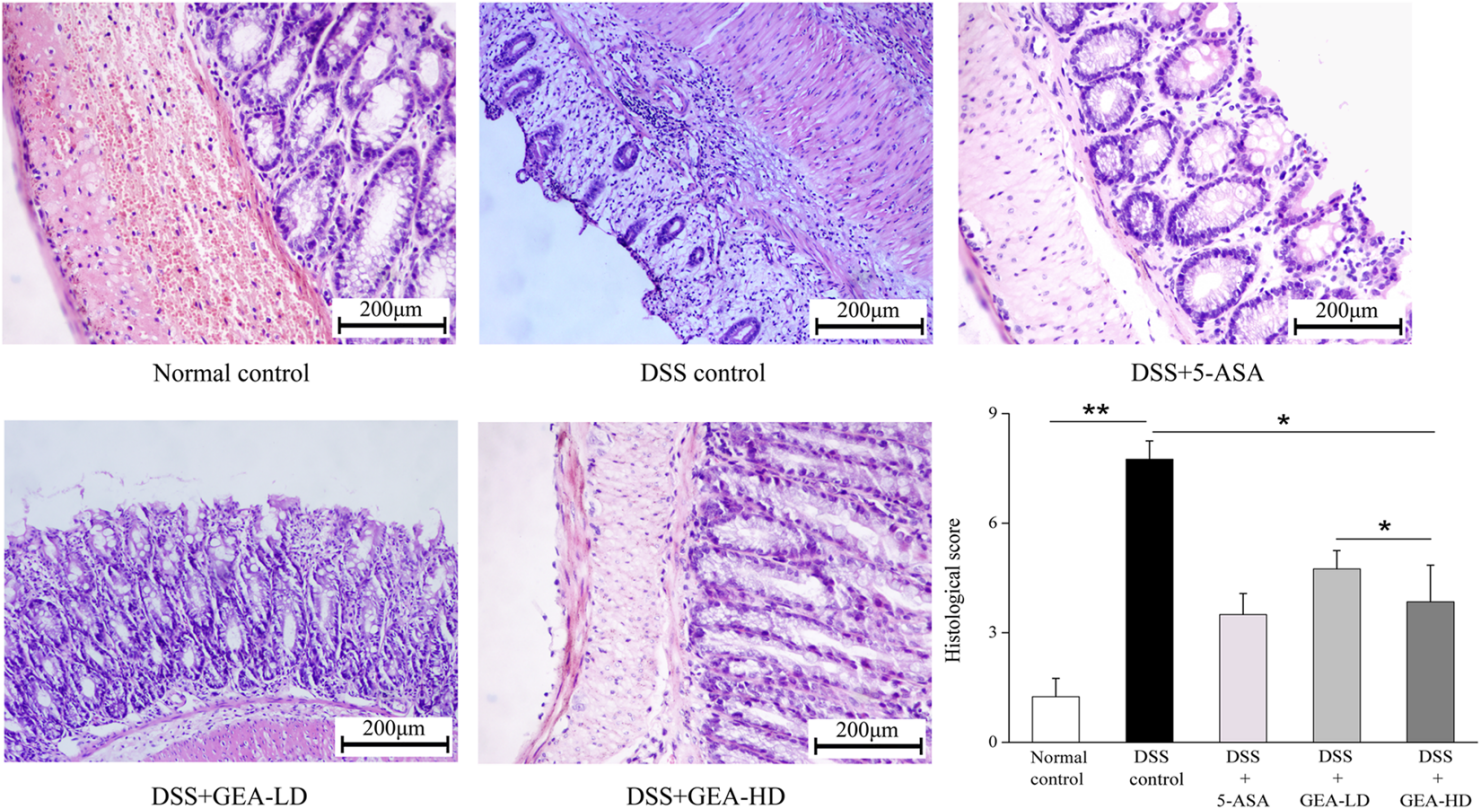
Turkish galls histologically inhibits DSS-induced UC in KM mice. The distal colon was stained by H&E-stained. Microscopic analysis shows that smaller and fewer colonic ulcers were detected in the GEA-LD group. This protected colonic epithelial cells from being damaged compared with the DSS control mice. The histological score was significantly lower in the Turkish galls group compared to the DSS group (P < 0.05). Scale bar = 200 μm. The values are expressed as the mean ± S.D. *P < 0.05, **P < 0.01, ***P < 0.001.

Pathological colon examinations were performed after H&E staining, and representative results are shown in Fig. 9. DSS-induced mice emerged with massive colon ulceration, severe inflammation, and crypt damage. After treatment with Turkish galls, tissue sections from representative areas of the colon showed that there was a decrease in crypts, as well as epithelial distortion. In the mucosal and submucosal areas, infiltrations of inflammatory cells, leading to significantly reduced histopathological scores on day 17 (P < 0.05), were observed.

#### Turkish galls suppresses the expression of MPO during DSS-induced UC

**Figure 10:**
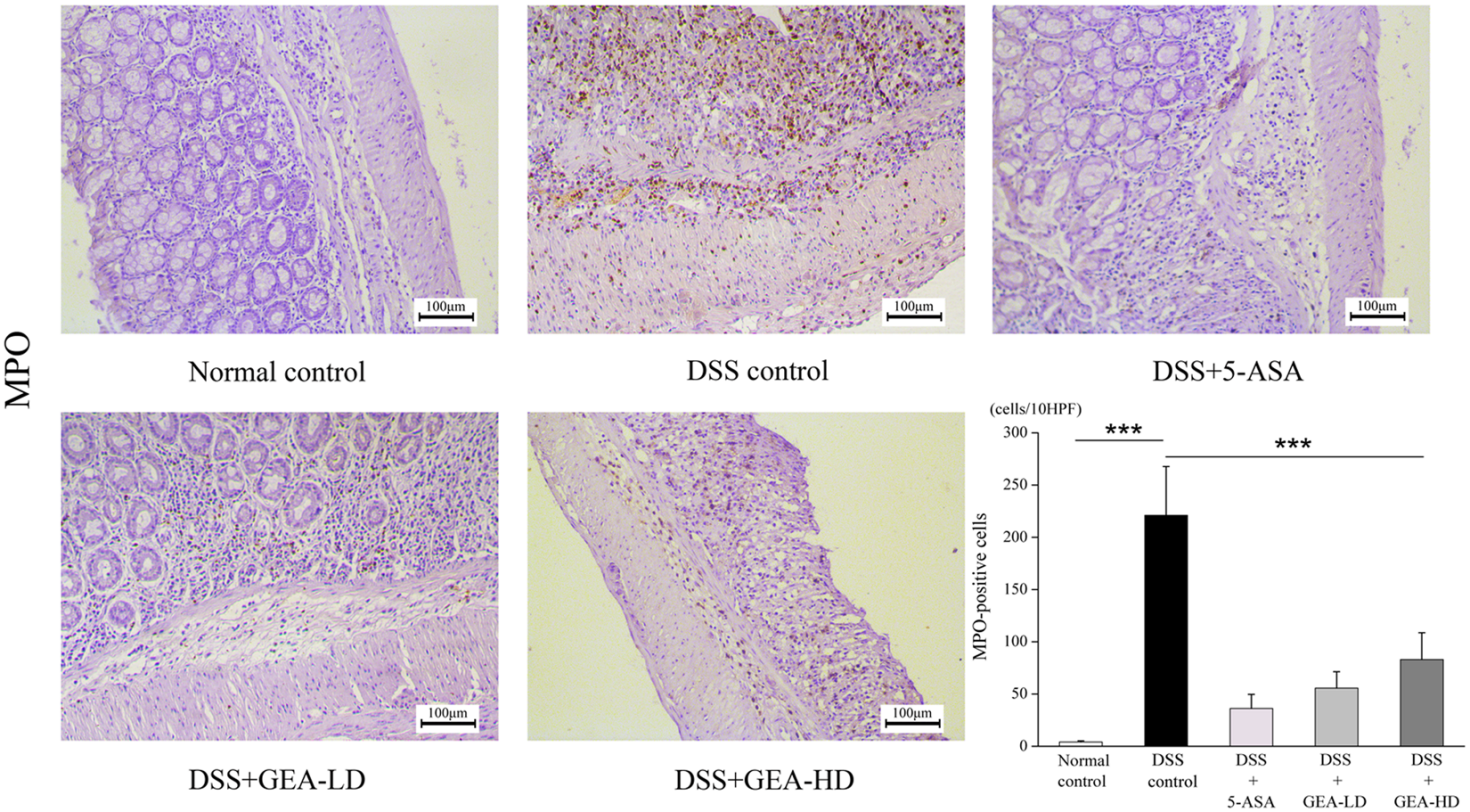
IHC for MPO showed that the numbers of accumulated neutrophils was significantly decreased in the GEA-LD and GEA-HD group compared with the DSS group. Scale bar = 100 μm. The values are expressed as the mean ± S.D. *P < 0.05, **P < 0.01, ***P < 0.001.

**Figure 11:**
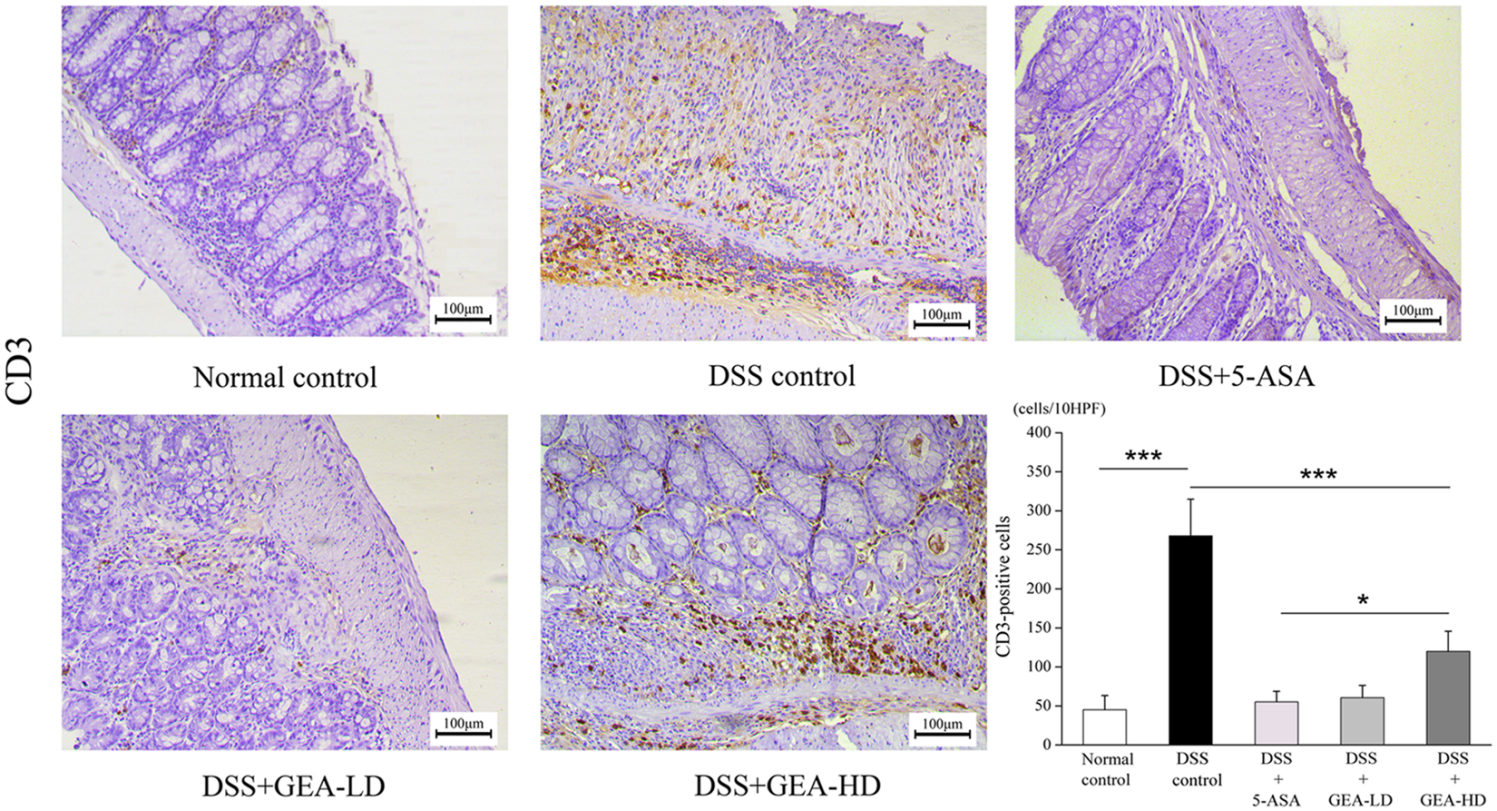
IHC for CD3 showed that the numbers of T-lymphocytes was significantly decreased in the GEA-LD and GEA-HD group compared with the DSS group. Meanwhile, the T-lymphocytes were higher in GEA-HD group compared with GEA-LD group. Scale bar = 100 μm. The values are expressed as the mean ± S.D. *P < 0.05, **P < 0.01, ***P < 0.001.

Myeloperoxidase (MPO) activity in colonic mucosa was measured as a marker of tissue neutrophil infiltration according to the following method with Immunohistochemistry (Fig. 10). MPO demonstrated that the MPO-positive cells (accumulated neutrophils) in the 5-ASA, GEA-LD and GEA-HD were significantly decreased than in the DSS group (P < 0.001). A fewer neutrophils in Normal control group was compared with DSS group (P < 0.001). IHC for CD3 demonstrated that the fewer infiltrating T-lymphocytes in the GEA-LD group compared with the DSS control group (P < 0.001). Meanwhile, we also found the T-lymphocytes were higher in GEA-HD group compared with GEA-LD group (P < 0.05) (Fig. 11).

#### Turkish galls suppresses the expression of cytokines levels during DSS-induced UC

**Figure 12:**
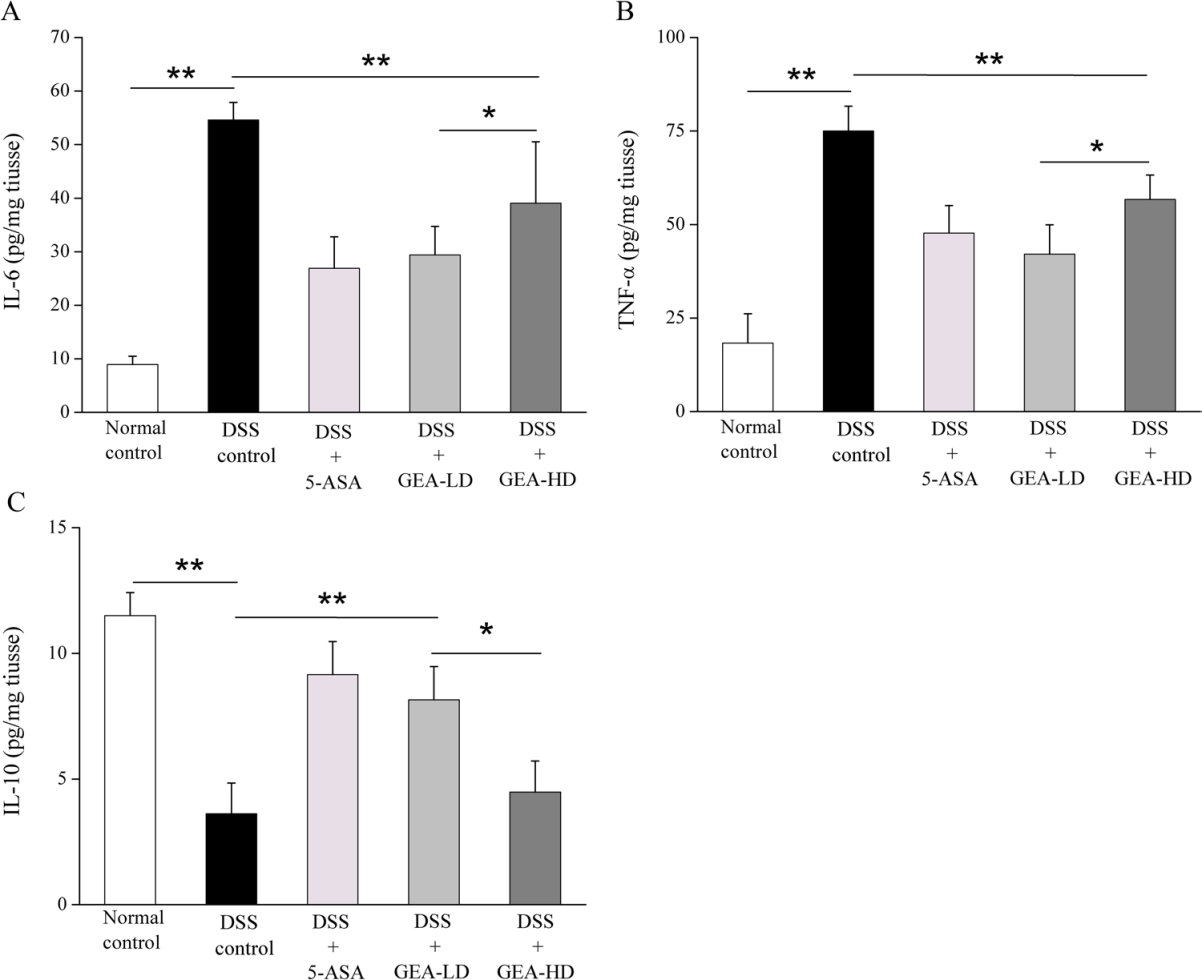
Turkish galls inhibits the expression of pro-inflammatory factors TNF-α, IL-6 and increases anti-inflammatory IL-10 in mice with DSS-induced UC. Turkish galls reduced the expression of inflammatory cytokines (**A**) IL-6, (**B**) TNF-α and increased the expression of (**C**) IL-10. An ELISA demonstrated that TNF-α and IL-6 were significantly higher in the DSS control group than in the GEA-LD/HD group (P < 0.01). IL-10 expression in DSS-induced mice was significantly enhanced by Turkish galls administration. The values are expressed as the mean ± S.D. *P < 0.05, **P < 0.01, ***P < 0.001.

Cytokines play an important role in the intestinal immune system. Immune cells, such as macrophages, dendritic cells, T cells, and intestinal epithelial cells, are known to secrete various cytokines. These cytokines regulate the inflammatory response in UC. Figure 12 shows the expression levels of inflammatory factor IL-10, TNF-α and IL-6 measured using ELISA. The expression of TNF-α and IL-6 was increased and IL-10 decreased in the mice treated with DSS, compared with the Normal control group (P < 0.01). After treated with Turkish galls, the expression of TNF-α and IL-6 was decreased and IL-10 was increased in the GEA-LD/HD group, compared with the DSS group (P < 0.01).

#### Effect of Turkish galls on immune cell subsets in the spleen

**Figure 13:**
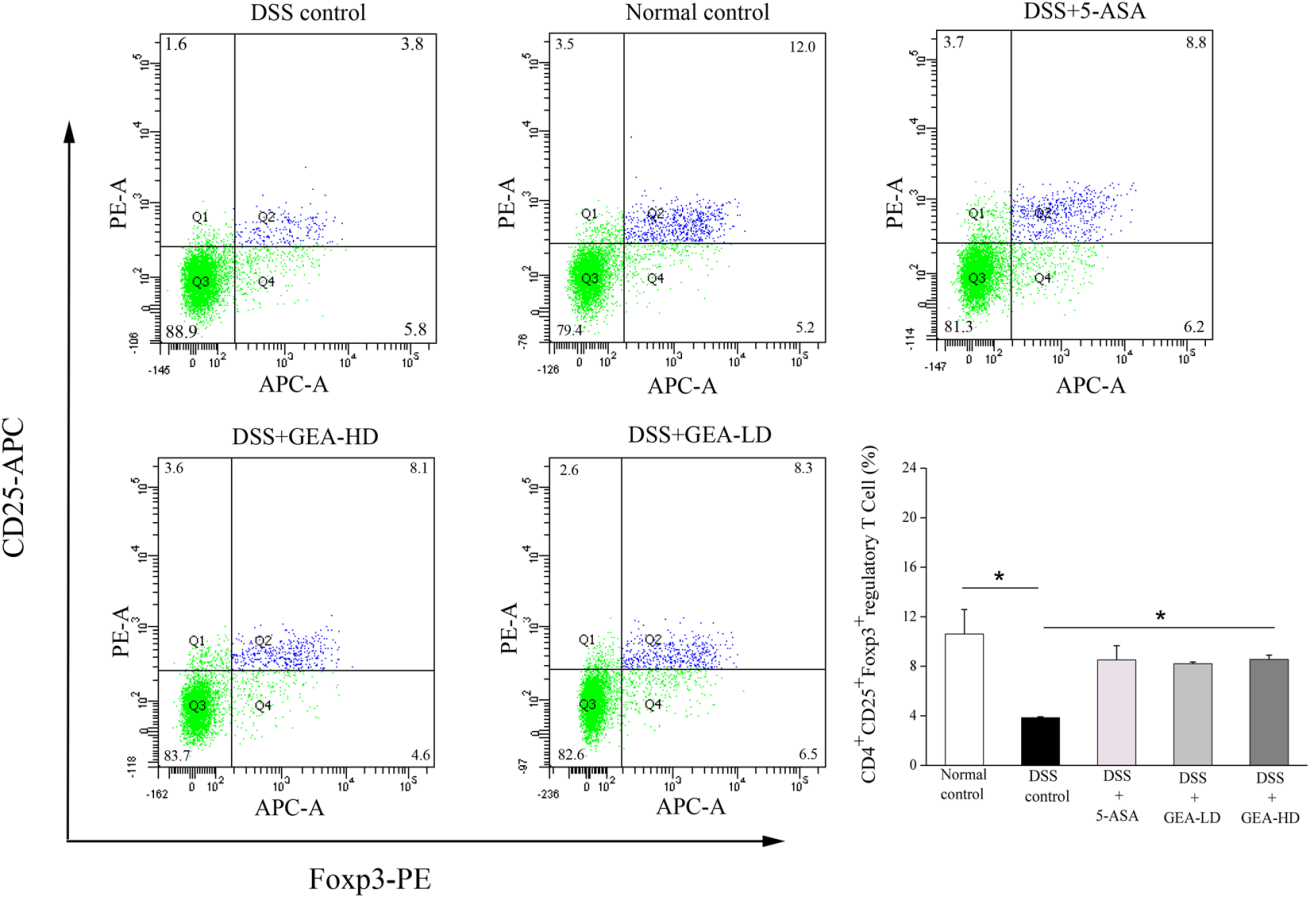
Turkish galls regulates the Treg cells in mice with DSS-induced UC. CD4^+^T cells isolated from SPs of mice after treated with Turkish galls. The number of Treg cells were measured using flow cytometric assay. The values are expressed as the mean ± S.D. *P < 0.05, **P < 0.01, ***P < 0.001.

To identify whether Turkish galls facilitates the differentiation of Treg cells, the lymphocytes were isolated from the SPs. They were stained with FITC-conjugated anti-CD4, APC-conjugated anti-CD25 and PE-conjugated anti-Foxp3 and the number of CD4^+^CD25^+^Foxp3^+^Treg cells counted using flow cytometry assay. There have been an increasing number of reports indicating that the immune imbalance of CD4^+^T cells plays a key role in the initiation and development of UC. CD4+ T cells are divided into 4 major subsets (Th1, Th2, Th17, and Tregs), which mainly function through the secretion of specific cytokines IFN-γ, IL-17, and IL-10. Moreover, Th17/Tregs and Th1/Th17 balances are critical for the treatment of UC^42–44^. Of these CD4^+^T cells, Treg cells are critical for the maintenance of immune homeostasis. They can regulate effector T cells and antigen-presenting cells (APC) by secreting anti-inflammatory cytokines, such as IL-10. This could significantly inhibit DSS-induced colitis in mice. IL-10-deficient mice spontaneously develop severe colitis^45,46^. In this study, IL-10 expression in UC mice colons was detected using ELISA. The data showed that the Turkish galls markedly increased the IL-10 level, and we also found that the administration of Turkish galls could significantly increase the number of Treg cells in UC mice SPs (Fig. 13). The data indicate that the anti-colitis effect of Turkish galls might be mediated by Treg cells induction and consequent increase in IL-10 in the colon.

#### Turkish galls treatment affects the mRNA expression levels of UC-associated targets by RT-PCR

**Figure 14:**
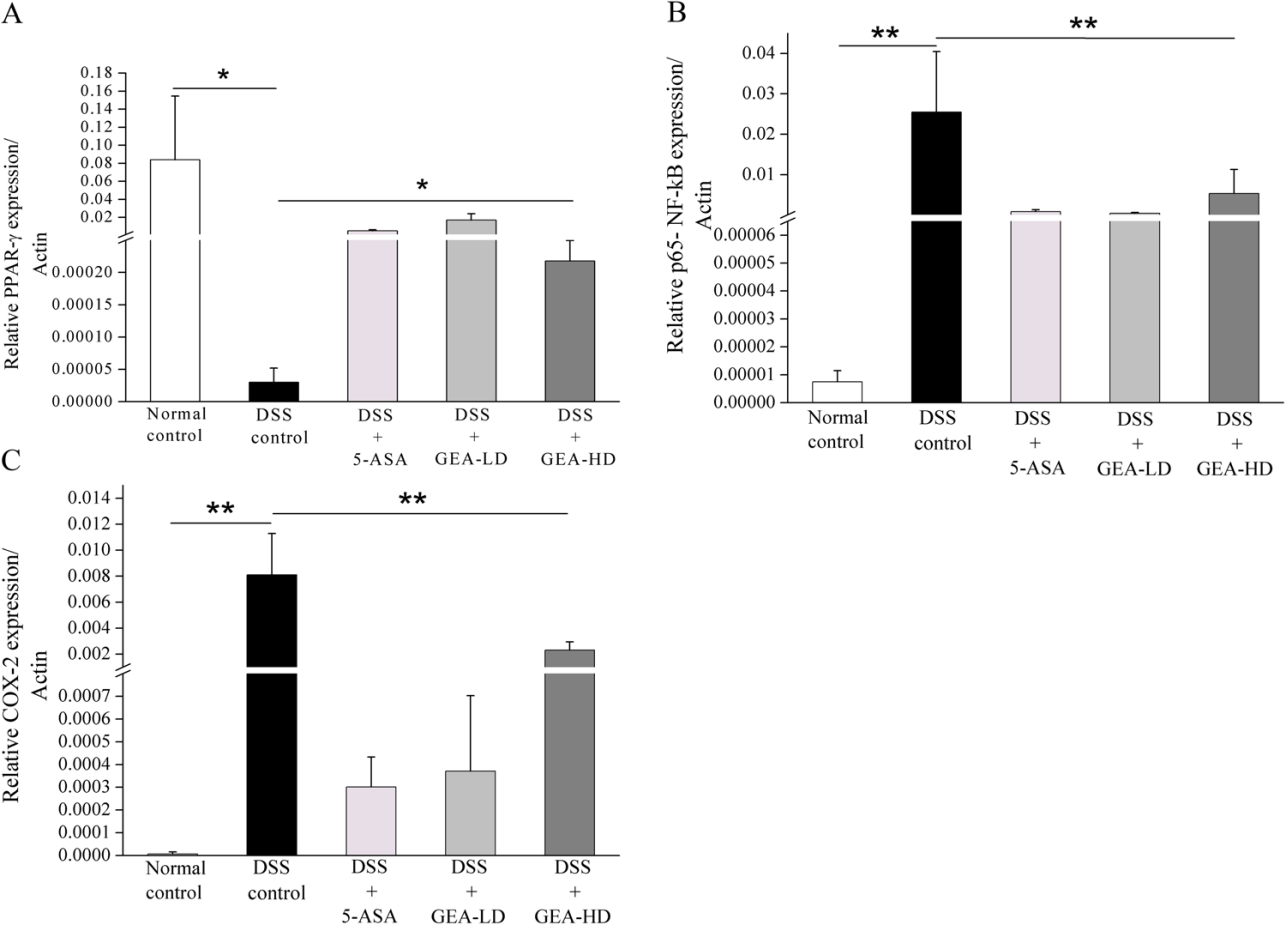
Turkish galls treatment affects the mRNA expression levels of UC-associated targets in mice. After 17 days, the mRNA expression levels of 3 genes were measured by RT-PCR. The relative amount of each gene was expressed using 2^−ΔCt^. The values are expressed as the mean ± S.D. *P < 0.05, **P < 0.01, ***P < 0.01.

To confirm our predictions of the molecular mechanism of Turkish galls experimentally, the PPAR-γ, COX-2, and p65-NF-κB were chosen for testing. Compared with normal control group, the expression of PPAR-γ was decreased in the DSS group (P < 0.05) (Fig. 14A), however, the expression of NF-κB and COX-2 were increased in the DSS group (P < 0.01) (Fig. 14B,C). After treated with Turkish galls, the expression of PPAR-γ was increased and the expression of NF-κB, COX-2 were decreased. The data indicated that the Turkish galls could activate the expression of PPAR-γ, attenuate NF-κB activation and inhibit the expression of pro-inflammatory cytokines and COX-2. Recent studies have shown that the PPAR-γ is a key inhibitor of colitis, by reducing NF-κB transcriptional activity^25,47–49^, or can interact with active p65 and facilitate its nuclear export in response to bacterial stimuli, which subsequently inhibits NF-κB activation^50^.

## Discussion

UC is a chronic, relapsing inflammatory bowel disease that predominantly implicates the large intestine. The etiology is unknown and deciphering the inflammatory mechanisms is difficult. KJA is a promising therapeutic for UC. However, due to its inherent features, such as multiple compounds and targets, its mechanism of action remains unclear. In this study, a new platform for system pharmacology was proposed, which evaluated the drug oral bioavailability, half-life, and target proteins, as well as the combined interactions of KJA for dissecting the addition and subtraction theory. The main results are summarized as follows:

1. Through ADME prescreening, 12 candidate compounds possessing favorable pharmacokinetic profiles were identified, in witch, 7 compounds were belong to tannis, and one compounds was phenolic acids. Meanwhile, the compounds of Turkish galls ethyl acetate fraction were identified by HPLC-ESI-MS/MS profiling analysis. The main components in Turkish galls were tannins representing about 88.14% of the total peak area. This result is consistent with the prediction made using system pharmacology. Twenty-two proteins targeted by these 12 compounds were identified as potential targets of the herb. These compounds and targets may contribute to guide our further research of Turkish galls.
2. By comparing and analyzing the C-T and T-D networks, 2 major conclusions were reached: (a) The therapeutic effects exhibited by Turkish galls in UC may be through 2 proteins, COX2 and PPAR-γ; (b) Through building and analyzing the UC pathway, we suppose that NF-κB pathways may be major signaling pathways, and several targets (including COX2 and PPAR-γ) are involved in NF-κB pathways, which are also critical targets of the C-T network. We assume that Turkish gall mitigates the severity of colitis by regulating the immune cell, Tregs, and inhibiting the expression of pro-inflammatory cytokines. Turkish galls also improved PPAR-γ expression, and attenuated NF-κB activation and subsequent COX-2 expression.
3. To validate those results, we provide evidence that Turkish galls extract has therapeutic effects in a mouse UC model. Pretreatment with Turkish galls showed overall less DSS-induced colonic damage, which was indicated by a reduced DAI and inflammatory histological score and an increase in colon length compared with DSS-induced colitis mice. We found that the NF-κB pathway is a major signaling pathway through building and analyzing the UC pathway, and the several targets (including COX2 and PPAR-γ) are involved in NF-κB pathways, Turkish gall mitigates the severity of colitis by improving the expression of PPAR-γ, leading to regulation of NF-κB control of the inflammatory response. COX-2, downstream of the NF-κB pathway, shows a similar response to Turkish galls treatments and a subsequent reduction in the expression of inflammatory factor IL-6 and TNF-α. MPO is linearly associated with the infiltration of inflammatory cells such as neutrophils, was used as a surrogate marker of the extent of inflammatory infiltration^51^. In our study, the significantly fewer infiltrating T-lymphocytes and neutrophil in colon tissue after treatment with Turkish galls. Therefore, these findings suggest that the anti-inflammatory effect of Turkish galls is likely to be associated with inhibiting the inflammatory cell infiltration, as well as the release of pro-inflammatory cytokines in colon tissue after DSS administration. These findings suggest that Turkish galls has potential therapeutic value in the treatment of UC. This result is consistent with the prediction made using system pharmacology.

In summary, this work has provided a deeper understanding of the pharmacological functions of Turkish galls from molecular level to systematic level, which may lead to further successful applications of systems pharmacology for TUM discovery and development.

## References

[1] Tanaka T (2011). Development of an Inflammation-Associated Colorectal Cancer Model and Its Application for Research on Carcinogenesis and Chemoprevention. International Journal of Inflammation.

[2] Lombardi VRM, Etcheverría I, Carrera I, Cacabelos R, Chacón AR (2012). Prevention of Chronic Experimental Colitis Induced by Dextran Sulphate Sodium (DSS) in Mice Treated with FR91. Biomed Research International.

[3] Sclano G (2002). Asthma, nasal polyposis and ulcerative colitis: a new perspective. Clinical & Experimental Allergy Journal of the British Society for Allergy & Clinical Immunology.

[4] Fang J (2013). Protection from inflammatory bowel disease and colitis-associated carcinogenesis with 4-vinyl-2,6-dimethoxyphenol (canolol) involves suppression of oxidative stress and inflammatory cytokines. Carcinogenesis.

[5] Yunusi K (2015). Uygur Medicine Xipayi Kui Jie’an Affects Gene Expression profiles in intestinal tissue Lesions in a Rat Model of Ulcerative Colitis. Bmc Complementary & Alternative Medicine.

[6] Committee CP. Drug Standards of the Ministry of Public Health of thePeople’s Republic of China. In: Book Drug Standards of the Ministry ofPublic Health of the People’s Republic of China. City: Uygur PharmaceuticalSection (1998).

[7] Aivazi AA, Vijayan VA (2009). Larvicidal activity of oak Quercus infectoria Oliv. (Fagaceae) gall extracts against Anopheles stephensi Liston. Parasitology research.

[8] Guo Xia Y, Kerim A (2009). Effect of Uyghur compound Xipayi Kuijie’an on the ultrastructure of small intestinal epithelial cell in Rat Model of Ulcerative Colitis. Journal of Xinjiang Medical University.

[9] Iminjan M, Yunus K, Hizbilla M, Hupur H, Li Y (2011). Experimental Study of Effects of Uygur Medicine Xipayi Kuijie’an on Colon Mucosa Apoptosis and the Mechanism of Treating Ulcerative Colitis. Keji Daobao/Science & Technology Review.

[10] Upur H (2011). The histologic effects of the Uyghur medicine xipayi kuijiean on ulcerative colitis in rats. Journal of the Australian Traditional-Medicine Society.

[11] Huang C (2013). Systems pharmacology in drug discovery and therapeutic insight for herbal medicines. Briefings in Bioinformatics.

[12] Li B (2014). Systems pharmacology-based approach for dissecting the addition and subtraction theory of traditional Chinese medicine: An example using Xiao-Chaihu-Decoction and Da-Chaihu-Decoction. Computers in Biology & Medicine.

[13] Ru J (2014). TCMSP: a database of systems pharmacology for drug discovery from herbal medicines. Journal of cheminformatics.

[14] Pei, T. *et al*. Systematic understanding the mechanisms of vitiligo pathogenesis and its treatment by Qubaibabuqi formula. *Journal of ethnopharmacology* (2016). 10.1016/j.jep.2016.06.001 27265513

[15] Zheng, C. *et al*. A novel systems pharmacology platform to dissect action mechanisms of traditional Chinese medicines for bovine viral diarrhea disease. *European Journal of Pharmaceutical Sciences* (2016). 10.1016/j.ejps.2016.05.018 27208435

[16] Ma C, Wang L, Xie XQ (2011). GPU accelerated chemical similarity calculation for compound library comparison. Journal of chemical information and modeling.

[17] Yu H (2012). A systematic prediction of multiple drug-target interactions from chemical, genomic, and pharmacological data. PLoS One.

[18] Smoot ME, Ono K, Ruscheinski J, Wang P-L, Ideker T (2011). Cytoscape 2.8: new features for data integration and network visualization. Bioinformatics.

[19] Sha T, Igaki K, Yamasaki M, Watanabe T, Tsuchimori N (2013). Establishment and validation of a new semi-chronic dextran sulfate sodium-induced model of colitis in mice. International immunopharmacology.

[20] Li Y (2010). Triptolide ameliorates IL-10-deficient mice colitis by mechanisms involving suppression of IL-6/STAT3 signaling pathway and down-regulation of IL-17. Molecular Immunology.

[21] Nishizawa M, Yamagishi T, Nonaka G, Nishioka I (1983). ChemInform Abstract: tannins and related compounds. part 9. isolation and characterization of polygalloylglucoses from turkish galls (quercus infectoria). Tannins and related compounds. XII. Isolation and characterization of galloylglucoses from Paeoniae Radix and their effects on urea-nitrogen concentration in rat serum. Chemical & Pharmaceutical Bulletin.

[22] Hasmida, M. N., Nur Syukriah, A. R., Liza, M. S. & Mohd Azizi, C. Y. Effect of different extraction techniques on total phenolic content and antioxidant activity of Quercus infectoria galls. *International Food Research Journal* (2014).

[23] Ordás I, Eckmann L, Talamini M, Baumgart DC, Sandborn WJ (2012). Ulcerative colitis. Lancet.

[24] Shrestha S (2014). Pharmacognostic studies of insect gall of Quercus infectoria Olivier (Fagaceae). Asian Pacific Journal of Tropical Biomedicine.

[25] Dubuquoy L (2006). PPARγ as a new therapeutic target in inflammatory bowel diseases. Gut.

[26] Jiang C, Ting AT, Seed B (1998). PPAR-γ agonists inhibit production of monocyte inflammatory cytokines. Nature.

[27] Rousseaux, C. *et al*. The 5-aminosalicylic acid (5-ASA) anti-neoplastic effect in the intestine is mediated by PPARγ. *Carcinogenesis* bgt245 (2013). 10.1093/carcin/bgt245 PMC3810841 23843037

[28] Bertin B, Dubuquoy L, Colombel J-F, Desreumaux P (2013). PPAR-gamma in ulcerative colitis: a novel target for intervention. Current drug targets.

[29] Desreumaux P (2001). Attenuation of Colon Inflammation through Activators of the Retinoid X Receptor (Rxr)/Peroxisome Proliferator–Activated Receptor γ (Pparγ) Heterodimer A Basis for New Therapeutic Strategies. The Journal of experimental medicine.

[30] Rousseaux C (2005). Intestinal antiinflammatory effect of 5-aminosalicylic acid is dependent on peroxisome proliferator–activated receptor-γ. The Journal of experimental medicine.

[31] Su, C. *et al*. In *Cytokines and Cell Homeostasis in the Gastrointestinal Tract: Proceedings of the Falk Symposium 113 Held in Regensburg, Germany*, *16–18 September, 1999*. 13 (Kluwer Academic Publishers).

[32] Feagan, B. G. & MacDonald, J. K. Oral 5 - aminosalicylic acid for maintenance of remission in ulcerative colitis. *The Cochrane Library* (2012). 10.1002/14651858.CD000544.pub3 23076890

[33] Lewis JD (2008). Rosiglitazone for active ulcerative colitis: a randomized placebo-controlled trial. Gastroenterology.

[34] Pedersen G, Brynskov J (2010). Topical rosiglitazone treatment improves ulcerative colitis by restoring peroxisome proliferator-activated receptor-γ activity. The American journal of gastroenterology.

[35] Roberts PJ, Morgan K, Miller R, Hunter JO, Middleton SJ (2001). Neuronal COX-2 expression in human myenteric plexus in active inflammatory bowel disease. Gut.

[36] Wang D, Dubois RN (2009). The role of COX-2 in intestinal inflammation and colorectal cancer. Oncogene.

[37] Rodríguez M (2014). Polarization of the innate immune response by prostaglandin E2: a puzzle of receptors and signals. Molecular pharmacology.

[38] Atreya I, Atreya R, Neurath M (2008). NF‐κB in inflammatory bowel disease. Journal of internal medicine.

[39] Spehlmann ME, Eckmann L (2009). Nuclear factor-kappa B in intestinal protection and destruction. Current opinion in gastroenterology.

[40] Wullaert A, Bonnet MC, Pasparakis M (2011). NF-κB in the regulation of epithelial homeostasis and inflammation. Cell research.

[41] Ren, T. *et al*. An adenosine A3 receptor agonist inhibits DSS-induced colitis in mice through modulation of the NF-κB signaling pathway. *Scientific reports* **5**, 10.1038/srep09047 (2015). 10.1038/srep09047 PMC4357005 25762375

[42] Glauben R, Sonnenberg E, Wetzel M, Mascagni P, Siegmund B (2014). Histone deacetylase inhibitors modulate interleukin 6-dependent CD4+ T cell polarization *in vitro* and *in vivo*. Journal of Biological Chemistry.

[43] Shi F (2012). Dysregulated Tim-3 expression and its correlation with imbalanced CD4 helper T cell function in ulcerative colitis. Clinical Immunology.

[44] Singh NP (2011). Activation of aryl hydrocarbon receptor (AhR) leads to reciprocal epigenetic regulation of FoxP3 and IL-17 expression and amelioration of experimental colitis. PLoS One.

[45] Malik A, Sharma D, St CJ, Dybas LA, Mansfield LS (2013). Contrasting immune responses mediate Campylobacter jejuni-induced colitis and autoimmunity. Mucosal Immunology.

[46] Owen CJ (2003). Mutational analysis of the FOXP3 gene and evidence for genetic heterogeneity in the immunodysregulation, polyendocrinopathy, enteropathy syndrome. Journal of Clinical Endocrinology & Metabolism.

[47] Chung SW (2000). Oxidized low density lipoprotein inhibits interleukin-12 production in lipopolysaccharide-activated mouse macrophages via direct interactions between peroxisome proliferator-activated receptor-gamma and nuclear factor-kappa B. Journal of Biological Chemistry.

[48] Hong R, Pownall HJ, Lodish HF (2003). Troglitazone Antagonizes Tumor Necrosis Factor-α-induced Reprogramming of Adipocyte Gene Expression by Inhibiting the Transcriptional Regulatory Functions of NF-κB. Journal of Biological Chemistry.

[49] Dubuquoy L (2003). Impaired expression of peroxisome proliferator-activated receptor γ in ulcerative colitis. Gastroenterology.

[50] Kelly D (2004). Commensal anaerobic gut bacteria attenuate inflammation by regulating nuclear-cytoplasmic shuttling of PPAR-gamma and RelA. Nature Immunology.

[51] Lin Y (2014). Transplantation of human umbilical mesenchymal stem cells attenuates dextran sulfate sodium-induced colitis in mice. Clinical & Experimental Pharmacology & Physiology.

