# Author Correction: A Systems Pharmacology Approach to Determine Active Compounds and Action Mechanisms of Xipayi Kuijie’an enema for Treatment of Ulcerative colitis

**DOI:** 10.1038/s41598-018-21978-7

**Published:** 2018-03-06

**Authors:** Wei Yu, Zhihong Li, Fei Long, Wen Chen, Yurong Geng, Zhiyong Xie, Meicun Yao, Bo Han, Teigang Liu

**Affiliations:** 1 School of Pharmacy, Xinjiang Shihezi University, Xinjiang, 832002 China; 2 The first affiliated hospital, School of medicine, Shihezi university, Xinjiang, 832002 China; 3 College of pharmacy, Sun yat-sen university, Guangzhou, 510006 China; 4 Key Laboratory of Chinese Internal Medicine of Education, DongZhiMen Hospital, Beijing, 100070 China; 5 College of Traditional Chinese Medicine, Beijing University of Chinese Medicine, Beijing, 100029 China

Correction to: *Scientific Reports* 10.1038/s41598-017-01335-w, published online 26 April 2017

In the original version of this Article, Teigang Liu was incorrectly listed as being affiliated with “Key Laboratory of Chinese Internal Medicine of Education, DongZhiMen Hospital, Beijing 100070, China”.

The correct affiliation is listed below:

College of Traditional Chinese Medicine, Beijing University of Chinese Medicine, Beijing 100029, China.

This has now been corrected in the HTML and PDF versions of this Article.

